# Support Seeking in the Postpartum Period: Content Analysis of Posts in Web-Based Parenting Discussion Groups

**DOI:** 10.2196/26600

**Published:** 2021-07-15

**Authors:** Bonnie R Chivers, Rhonda M Garad, Lisa J Moran, Siew Lim, Cheryce L Harrison

**Affiliations:** 1 Monash Centre for Health Research and Implementation School of Public Health and Preventive Medicine Monash University Clayton Australia; 2 Diabetes and Vascular Medicine Monash Health Clayton Australia

**Keywords:** pregnancy, perinatal, maternal, postpartum, infant, social support, qualitative, health, online

## Abstract

**Background:**

The transition from pregnancy to motherhood is a major developmental phase that can be challenging for both women and their families. For new mothers, the postpartum period is recognized as a critical period for increased risk of both physical and mental health concerns. For this reason, it is imperative that women receive accurate, evidence-based information during this time.

**Objective:**

This study aims to explore the conversations of new mothers on a web-based parenting forum to investigate what topics or concerns are being discussed.

**Methods:**

A leading Australian web-based support forum for women before and after birth was used to obtain a sample of posts from the mothers of infants aged 0-12 months. Quantitative data (word frequencies and sentiment analysis) and qualitative data (post content) were extracted from discussion threads and examined to determine sentiments and theoretical storylines.

**Results:**

In total, 260 posts were sampled. Infant care was the most prominent overarching topic discussed, with feeding and sleep being the most discussed subtopics. Discussions about maternal care were much less frequent but included questions about birth recovery, breastfeeding concerns, and interconception. A pattern of behavior emerged within the posts. This pattern resembled a cycle of learning across five phases: help seeking, solution ideation, testing and skill development, consolidation, and empowerment and improved mental well-being. A dynamic interplay was observed as mothers navigated new concerns or developmental changes.

**Conclusions:**

Engagement in web-based forums to seek help and support during the postpartum period was common, with infant health and well-being being the primary concerns for new mothers during this time. The identification of a maternal learning cycle within the forum underscores the contributory role of web-based communities in maternal peer social support, information seeking, and early parenting practices.

## Introduction

The transition from pregnancy to motherhood is a major developmental phase that is recognized as a challenging time for both women and their families [1]. Early parenting and infant care are often prioritized over the health of the mother, presenting as significant barriers to self-care in the early postpartum period [2-4]. These barriers inhibit efforts to maintain or improve overall health, mental health, and healthy lifestyle behaviors [2-4], such as adequate diet quality and regular physical activity [5]. For these reasons, the postpartum period is recognized as a critical period for an increased risk of adverse health. Weight retention after pregnancy is common [6,7] and is associated with excessive gestational weight gain [8], which is a strong predictor for the development of future obesity and chronic diseases [7]. Furthermore, mental health disorders, including anxiety and depression, affect up to 20% of women following pregnancy [9,10]. Postpartum anxiety and/or depression can exert significant effects on the health and well-being of mothers, their partners, and other children and can exert a negative impact on infant development [11]. Therefore, given the vulnerability to adverse physical and mental health, new mothers are a unique population with specific health needs that require increased support as well as accurate and trustworthy health information and care.

During the postpartum period, almost three-quarters (73%) of Australian parents with children aged less than 5 years use websites, blogs, and web-based forums to obtain information about infant or child health and parenting [12-14], with similar findings reported internationally [15]. Australian households have a considerably high internet use, with 97% of households with children having internet access [16]. Previous research evaluating the drivers of internet use during this time reported convenience, anonymity, and social and peer support as facilitating factors [13,17,18]. Web-based parenting forums are a common platform in which women can connect with peers for emotional support; alleviate feelings of isolation; and facilitate the discussion of sensitive topics that are otherwise difficult to address with friends, family, or health care providers in face-to-face encounters [18]. Yet, although reasons for engagement are clear [1,12,13,18], there is limited evidence showing what information and support women seek during the postpartum period and how they interact within such forums. An improved understanding of the information and support needs of women during this significant life phase is crucial to ensure that health and information needs of the mothers are met.

To address this research gap, this paper examines the unmediated user-generated content from web-based forum discussions of women in the postpartum period to identify early parenting information and emotional support–seeking behaviors.

## Methods

### Overview

An observational analysis of web-based discussions was conducted within a leading Australian internet discussion forum for new or expecting parents. The most popular Australian pre- and postbirth forum was identified by searching the term *new mum forum* on Google. The top 10 (first page) results were assessed, and all the websites with publicly available forums (n=7) were analyzed using a website analytics tool (Alexa, Amazon). This software was used to determine the global page views, global rank, and Australian rank of the seven websites with publicly available forums. The highest ranked website for Australian users was identified and used as the sampling platform for this study. To confirm the suitability of this website for this study, member requirements were assessed to ensure that forum users were new or expecting mothers. The second highest ranked site did not have an open access forum, and those ranked 3-10 had a significantly lower rank than the first and second highest ranked sites. Therefore, the first site was retained, with the remainder deemed insufficient for sampling. This approach is comparable with previous research that used discussion forums [19,20].

The selected forum allows members to interact with their *birth club*, which corresponds to their child’s due date (month and year). Birth clubs are a subforum of the wider forum community, and they are nonspecific in the nature of discussion topics. In total, 13 birth clubs (January 2019 to January 2020) were selected as the sampling platform to represent 1 calendar year and therefore one cross-section of the postpartum period across this time (ie, <1 month to 12 months postpartum).

Included posts were sampled by selecting the first 20 posts or threads from each birth club at the time of collection. Posts were collected between January 6, 2020, and January 13, 2020. The exclusion criteria included posts that enquired about or discussed an elder sibling (not the infant aged 0-12 months), other people’s child or children, extended family such as grandparents or in-laws, or products or shopping (unless the post also discussed infant care such as feeding product advice). Posts in the January 2020 birth club were excluded if the forum user indicated that they had not yet given birth. Posts were collected sequentially, as they appeared on the day of sampling, and if a post met the exclusion criteria, the following post was selected until 20 posts were obtained. Posts were extracted in a deidentified format into an Excel (Microsoft) document that included the post title, date, and content, comparable with previous research [19,20].

### Analysis

Data were processed using NVivo Pro 12 software (QSR International) [21]. A modified grounded theory analysis was conducted, which was informed by the six-phase approach by Braun and Clarke [22]. Due to the understudied nature of parenting forums, a grounded theory approach is well suited to add depth and breadth to this investigation [23]. A single researcher (BRC) generated initial codes and then grouped them into core categories. Three authors agreed on initial and intermediate codes and conducted a narrative overview of the discussions (BRC, RMG, and CLH). A >10% (26/260) check was conducted after initial coding and during theme conceptualization by 2 additional researchers (RMG and CLH). As the themes were conceptualized, the research team developed a theoretical storyline, which could be observed beyond *what* was being discussed.

To support these findings, NVivo Pro 12 text frequency search was used to identify the common terms, thereby identifying prominent conversation topics. Word frequency calculations identified all stemmed words (minimum three letters). NVivo Pro 12 automatic sentiment analysis was performed to identify the emotional indicators. NVivo searched for the expressions of sentiment in the source material (forum posts) and used predefined scores for words classified as containing sentiment [24]. Words are considered in isolation, and the program then determines the sentiment of the paragraph as a calculation of each word containing the sentiment. Sentiment results include the number of references (paragraphs with sentiment) that are categorized as very positive, moderately positive, moderately negative, and very negative [24].

### Ethics

The Monash Health (RES-19-0000-291A) and Monash University (project no. 20196) Human Research Ethics Committees granted ethics approval for this study. Although the ethical oversight of publicly available data is not strictly required, the authors sought approval as per the Monash University protocol.

## Results

### Overview

In total, 260 posts were extracted and analyzed. The 13 birth clubs had an average of 3013 members in each club (n=39,163 forum members overall).

### Analysis of User-Generated Content

The analysis of posts through open coding identified 432 references at the intermediate coding stage. Various posts discussed multiple topics; therefore, the number of topic references exceeded the number of posts. Infant-focused references were the most frequent (237/432, 54.8%), with 12.5% (54/432) of references relating to sleep and naps. References to infant health (46/432, 10.6%) and feeding were frequent (68/432, 15.7%), and 7.6% (33/432) of references were related to breastfeeding. Forum use to seek help, support, advice, or reassurance was frequent (71/432, 16.4%). Discussion topics relating to infant care commonly centered on health (eg, nappy rash, cracked lips, and cradle cap) and development (eg, common milestones including teething and sitting, crawling, or walking). Both first time and mothers with older children were active in these discussions. Women regularly used the forum to ease concerns and to assist them in times of need or confusion during their first year of motherhood.

Maternal health needs and/or well-being were less frequent, with the overall identification of 21.1% (91/432) of references. Most maternal health discussions were observed in the early postpartum period and became less frequent further on from the birth experience. Topics pertaining to maternal health included birth recovery, breastfeeding difficulties, mastitis or breast discomfort, pelvic floor health, and resumption of menstruation. There was limited discussion about modifiable health factors, including the mother’s weight, exercise, or diet. The evidence of mental distress was observed with some women discussing the feelings of anxiety, birth trauma, or unhappiness (Table 1).

The discussion forum was used in tandem with care or advice from health professionals, not in place of it. Women appeared to use the forum to confirm a health issue, seek out the experiences of other mothers, or share their experiences. There was no evidence of disregarding the health advice from the health professionals.

Mothers reached out to other forum users when they were unsure about how to manage something and sought a similar experience from others in their birth club commonly asking the following: “Has anyone else had this?,” “Has anyone else been told this?,” “Can anyone else relate?,” and “Anyone going through the same thing?” These inquiries match efforts to normalize experiences or to confirm a problem. Mothers were often observed describing a problem to ask others if this was *normal* and to determine if they should seek advice from health professionals: “Should I be concerned?,” “What do I do? Should I take him to the doctor?...or is this normal?,” and “Is this something I should be worried about at this age.” Some mothers used the forum to allay worries as they bridged time until they could reach their doctor: “I am taking him to the doctors tomorrow but I just wanted to know if anyone has experienced this.”

**Table 1:** Intermediate coding references (N=432).

Topic, subthemes, and references					Codes, n (%)
**Infant**					237 (54.9)
	**Sleep**			54 (12.5)	
		Daytime or nighttime sleep routine	30 (6.9)		
		Bad sleeper	5 (1.1)		
		Hunger and sleep relationship	4 (0.9)		
		Cosleeping	3 (0.7)		
		Sleep regression or changes	4 (0.9)		
		Sleep training	3 (0.7)		
		Clothes or swaddle for sleep	2 (0.5)		
		Not sleeping due to teething	2 (0.5)		
		Sleep safety	1 (0.2)		
	**Infant health**			46 (10.6)	
		Skin concerns or topical treatments	15 (3.5)		
		Miscellaneous	12 (2.7)		
		Weight concerns	4 (0.9)		
		Bowel movements	4 (0.9)		
		Immunizations	4 (0.9)		
		Blood or mucus in nappy	3 (0.7)		
		Common cold	2 (0.5)		
		Tongue tie	2 (0.5)		
	**Routines**			39 (9)	
		Daytime nap routine	15 (3.5)		
		Nighttime sleep routine	15 (3.5)		
		Feeding routines	9 (2.1)		
	**Feeding**			35 (8.1)	
		Feeding solids	12 (2.7)		
		Formula amount or recommendations	6 (1.4)		
		Feeding routines	6 (1.4)		
		Refusing bottle	3 (0.7)		
		Unusual food-related behavior	3 (0.7)		
		Feeding cow’s milk	2 (0.5)		
		Unable to burp or upset tummy	2 (0.5)		
		Dad wanting to help feed	1 (0.2)		
	**Breastfeeding**			33 (7.6)	
		Milk supply	20 (4.6)		
		Breastfeeding routines	3 (0.7)		
		Pain or discomfort	3 (0.7)		
		Breastfeeding in subsequent pregnancy	2 (0.5)		
		Weaning	2 (0.5)		
		Drinking alcohol and breastfeeding	1 (0.2)		
		Feeding aides, that is, shields	1 (0.2)		
		Number of breastfeeds	1 (0.2)		
	**Development**			19 (4.4)	
		Teething	7 (1.6)		
		Leap	5 (1.1)		
		Crawling	3 (0.7)		
		Talking	2 (0.5)		
		Walking	2 (0.5)		
	**Infant behavior**			6 (1.4)	
		Behavioral problems or concerns	6 (1.4)		
	**Miscellaneous**			5 (1.2)	
		Haircuts or ear piercing	2 (0.5)		
		Travel	2 (0.5)		
		Car seats	1 (0.2)		
**Maternal**					156 (36.1)
	**Help seeking**			71 (16.4)	
		Seeking emotional support	15 (3.4)		
		Seeking advice or reassurance	56 (12.9)		
	**Psychosocial health**			30 (6.9)	
		Emotional well-being	17 (3.9)		
		Anxious about something	4 (0.9)		
		Feeling lost or guilty	4 (0.9)		
		Lack of support	3 (0.7)		
		Changes and challenges	2 (0.5)		
	**Interconception**			25 (5.8)	
		Sleep deprived	6 (1.4)		
		Menstrual cycle returning	6 (1.4)		
		Subsequent pregnancy announcement	6 (1.4)		
		Becoming pregnant again (views or concerns)	4 (0.9)		
		Birth control	2 (0.5)		
		Irregular periods	1 (0.2)		
	**Birth recovery or physical health**			24 (5.6)	
		Natural birth recovery	12 (2.8)		
		C-section recovery	4 (0.9)		
		Weight loss	4 (0.9)		
		Birth experience	2 (0.5)		
		Stretch marks	1 (0.2)		
		Pelvic floor	1 (0.2)		
	**Socializing**			6 (1.4)	
		Networking	6 (1.4)		
**Medical**					39 (9)
	**Health provider advice**			23 (5.3)	
		Querying the advice of health provider (general physician or maternal child health nurse) with forum community	13 (3)		
		Discussing concern before seeking professional advice	10 (2.3)		
	**Questioning**			16 (3.7)	
		Clinically relevant questions	16 (3.7)		

### Overview of Advanced Coding

**Figure 1:**
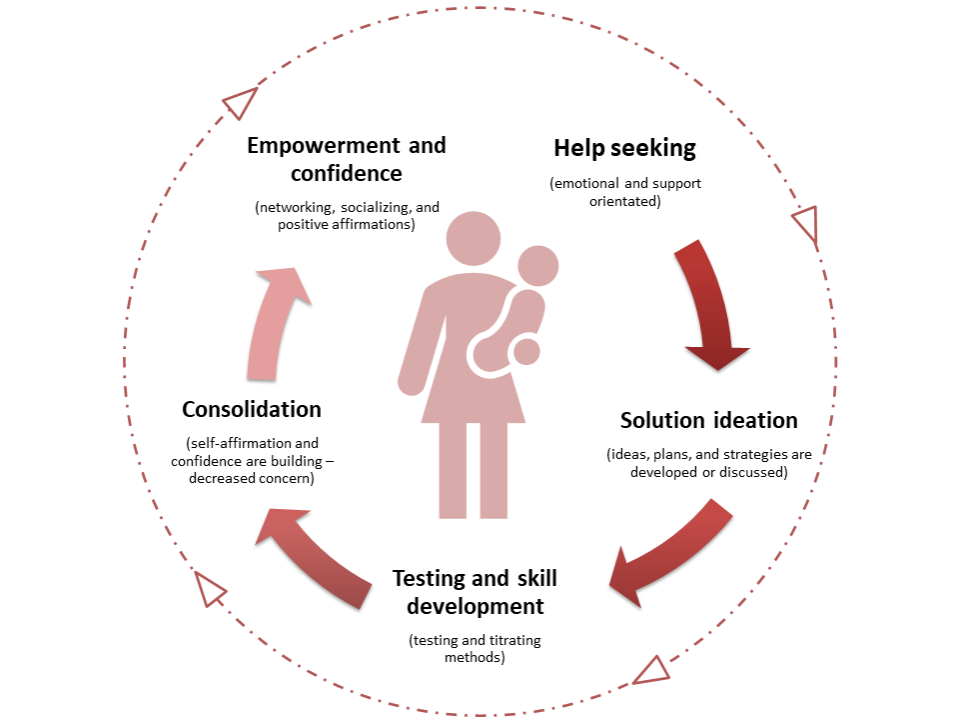
Schematic of the learning cycle.

An emerging cross-cutting pattern through posts was observed while examining web-based discussions in the cohort. Although the discussion topics identified remained consistent, the mother’s approach to discussing these concerns, how the concern was expressed, and level of support sought varied. This pattern was reflective of a cycle of learning across five phases: (1) help seeking, (2) solution ideation, (3) testing and skill development, (4) consolidation, and (5) empowerment and improved mental well-being (Figure 1). As mothers moved through the stages of parenting and learning, confidence among forum members increased and confusion, uncertainty, and the need for reassurance decreased. During the final stages of the learning process, women often presented as more empowered and self-assured, with some sharing stories of reassurance and others assuming the role of peer support providers to mothers in the early postpartum period. Although we describe a process model, in practice, we believe that early parenting experiences and parental skill development by mothers are characterized through a dynamic interplay within the model. Mothers may interact with different phases simultaneously, as they encounter new challenges that cause stress and uncertainty; yet, they are further progressed and more confident in other aspects of early parenting. For example, a mother may feel confident about infant feeding (later phases) and yet be anxious and uncertain about how to manage teething (earlier phases).

There appeared to be a temporal relationship between the length of time parenting and increased confidence. This is presumed and cannot be confirmed by this study; however, early postpartum mothers expressed greater levels of uncertainty and concern than those interacting with the forum during the later postpartum period. Overall, the vast majority of posts were from women expressing phases one or two, women who portrayed distress and uncertainty, and women who were more likely to seek support and advice from others. A minority of posts belonged to the later phases of the learning cycle, indicating that women were potentially more reassured and less inclined to engage with the forum specifically to share positive experiences.

### Five-Phase Learning Process (Drawn From Advanced Coding)

#### Phase One: Help Seeking

Women using the forum initiated discussions on an issue, need, or problem relating to their child or children or parental experiences. Conveying emotional experiences was common in this phase, as was maternal uncertainty. Women regularly stated that they were feeling *anxious*, *stressed*, *worried*, or *exhausted*. The forum acted as an outlet for these emotions and an opportunity to receive social and emotional support from other women:

our little miss is the worst sleeper ever [...] I am up and down all night we are lucky to get 3 hours [...] please any book ideas, throw them at me. I’m physically, emotionally and mentally drained.

I’m really down at the moment [...] My 8-month-old doesn’t sleep through, she doesn’t show me any affection and is quite sooky and demanding. I [...] just feel like I’m waiting for the stage to pass so I can be happier and feel some sort of motivation.

my little one is 9 weeks old and I still feel so clueless, does anyone else? [...] I just feel a bit lost.

In the early postbirth period, many women used this stage to discuss their experiences before seeking health professional’s advice or while bridging the time until they can seek help:

[I have the symptoms of an] episiotomy hematoma. Has anyone had any experiences with this? I’m checking in with my OBGYN tomorrow.

#### Phase Two: Solution Ideation

Following the initial requests for help and/or information, women discussed potential solutions. Discussing or testing strategies or ways to resolve their concerns with their peers in the forum was common to this phase:

I need your help. My poor little baby is super constipated [...] Nothing has worked! I have tried pear and prunes, water, pear juice, Coloxyl, brown sugar and water.

Solution ideation was also used by women building confidence to make changes:

for those of you who have started snacks for baby. What are you offering? Need ideas. Also breastfeeding mamas do you still offer a boob [breast] feed before putting baby down for a nap?

#### Phase Three: Testing and Skill Development

In this phase, women started to implement strategies and test solutions. Using a trial-by-error approach, mothers titrated methods to obtain the best outcomes related to their concern. They discussed their results with their peers while seeking reassurance and guidance during this process:

recently increased my 16-week-old to 150ml and 5 bottles [...] she’s struggling to take even 90ml at a time?! I [have] stretched to 4 hours thinking she might not be hungry but still no difference working. [...] she missing out on around 300 of the total, should I be worried? she’s seems her usual happy self maybe napping a little more.

During this phase, women have the confidence to try things or rationalize their experiences, yet require reassurance from their peers:

...is that too ambitious even for an 8-month-old? Does anyone else’s baby not babble at all at 8 months? and/or what sounds are your babies making by now?

Mothers often seek insight from the past experiences of others or their peers with a child of comparable age.

#### Phase Four: Consolidation

During this phase, women consolidated their new skills and practices. This phase was often coupled with an increased confidence and decreased uncertainty. It was common for women to post step-by-step outlines of their daily routines to compare with those at a similar stage. Women who anticipated that they were approaching a successful outcome were seen to reaffirm what they had tried or achieved, such as:

breastfeeding has never been easy, [...] however I was told to just persist. [...] He is already feeding a lot better than he was previously. Fingers crossed it gets better and better so I can go back to exclusively breastfeeding.

A common process observed during this phase was the consolidation of advice received from health professionals:

she said that from 9 months, milk is secondary and food is to be offered first always. Has everyone else been told this? I’ll follow her advice. Was just checking.

#### Phase Five: Empowerment

In this final phase, women displayed a degree of empowerment characterized by an increased confidence in the use of acquired knowledge or skills. Many assumed the role of information provider to other mothers, which could be viewed via the responses to original posts, characterizing somewhat of a team working together to share ideas and support those in need. Within the original posts, we viewed women at this stage reaching out for connections, such as “how are all the mummas doing? just checking in,” or networking “any mums living close to [...] who would like to connect.” Those with the confidence to do so shared their experiences to support and guide others:

I wanted to create a thread in case you’re feeling a bit down and want some encouragement from other women navigating their first/second/third/tenth time through the postpartum recovery journey. [...] It’s not pretty, let’s say that. [...] I’m tired but [...] I’m wandering around like an elderly lady, blissfully happy with our third born child [...] and feeling the pains, irks and exhaustion.

### Sentiment and Word Frequency Analysis

A word frequency calculation supports the findings of our open coding with *baby*, *sleep*, *feeds*, three of the five most frequently used words (Multimedia Appendix 1). There were 335 references of sentiment. Of these, most were found to be within the negative range, with 64.2% (215/335) of references classified as negative (very negative: n=126 and moderately negative: n=89) compared with 120 positive references (very positive: n=48 and moderately positive: n=78). A cross-check of sentiment results revealed that less than <10.1% (34/335) of the sentiment references were coded as incorrect categories.

## Discussion

### Principal Findings

This observational study examined unmediated peer-to-peer web-based discussions during the postpartum period, providing important insights into the information- and help-seeking needs of new mothers. We used the largest digital platform for new parents in Australia, which was representative of approximately 9.8% (30,000/305,832) of women giving birth annually [25], in line with engagement to opt in survey-based methods evaluating health behaviors [26]. Our results demonstrate a predominant focus on infant health needs, including feeding, breastfeeding, and sleep, during early parenting, with maternal health and well-being being a minor focus. Sentiment analyses revealed that the posts were more likely to be negatively portrayed, supporting the finding that the forum is commonly used to express a problem, seek information or help, and gain support or reassurance, consistent with previous literature [20]. The thematic analysis of posts revealed a pattern of behavior resembling a learning process whereby topics remained consistent, but how the concern was expressed and the level of support required varied. This process revealed several phases that commenced with help seeking through consolidation and empowerment. Overall, our findings provide critical insight into the concerns of new mothers and underscore the contributory role of web-based communities in maternal peer social support, information seeking, and the development of early parenting practices.

We report a central focus around the care and development of the infant, with a minority of posts about maternal well-being and fewer again centered on preventive health, including weight gain prevention or modifiable lifestyle factors. This finding is significant, given that the risk of adverse health is high, suboptimal lifestyle behaviors and weight gain are common, and barriers to health optimization exist during the postpartum period [2-4]. Previous studies have reported that time constraints are the most prominent barrier to healthy lifestyle changes, including physical activity engagement, at both 3 and 12 months after pregnancy [3]. In addition to reaffirming these barriers, our results provide additional insights and findings that personal health and well-being were not prominently discussed by mothers, suggesting that this is not a central priority during early parenting compared with that of infant health. This is potentially reflective of reduced engagement and compliance in postpartum healthy lifestyle interventions, as reported previously [27]. Subsequently, there is a paucity of effective strategies to engage women during this life phase for optimized health. Taken together, this emphasizes the need for novel approaches to enhance the awareness of, and engagement in, maternal preventive health during this period. This could potentially include maternal healthy lifestyle promotion delivered alongside infant care or design of holistic lifestyle programs including infants and wider family members to improve feasibility and engagement for new mothers. Alternatively, the implementation of healthy lifestyle programs during pregnancy when women are regularly engaged with health care providers has been shown to increase compliance in the postpartum period [28] and may better optimize lifestyle behaviors if practiced and maintained before birth.

Our results identified that discussion themes were underpinned by a learning development process, not dissimilar to those previously described, such as Becoming a Mother and Maternal Role Attainment theories [29-31]. The findings of this study emulate the concepts outlined in previous theories regarding maternal development (psychological adjustments and acquisition of a new role; acquaintance, learning, and physical restoration, which are both assumed and learned; and internalization of the maternal role, competence, and confidence). The crucial developmental processes at play within the forum emphasize the importance of the internet during this period and illustrate the influence forum communities may have on maternal decision-making and experiences. Lupton [18] previously identified that women use forums and web-based social networks to connect with other women and to gain guidance and insight through others’ experiences and knowledge. We note that the majority of posts were in the early phases of the learning cycle, in which mothers were uncertain, requiring an increased emotional support or solution seeking from their peers. This is also reflected in the sentiment analysis results, with higher proportions of negative sentiment compared with positive sentiment. Our results show that an increasing maternal confidence potentially coincides with the skill and knowledge acquisition sought within web-based communities. Although not all knowledge and skills may be obtained through the forum, and the de-escalation of stress and uncertainty may be influenced by many factors, the forum community is clearly an important platform during early parenting knowledge acquisition for many women and, therefore, plays a significant role in the postpartum journey for new mothers.

Supporting parents to meet the challenges of their caregiving role has consistently been identified as a public health priority [32,33]. Despite this widespread recognition [34], knowledge gaps still exist regarding effective ways to promote positive parenting practices, and little evidence is available that clearly depicts how parents learn and develop [32]. Ensuring health professionals are aware of the support requirements of women during this phase as well as their information priorities, as identified here, is essential. Furthermore, the understanding that acquisition of knowledge and skills during early parenting is fluid and follows a learning cycle is important in enabling the provision of individualized information and support. Similarly, assisting women in recognizing these learning processes may alleviate postpartum stress [35] and anxiety experienced during the initial phases and, in turn, facilitate more rapid progression toward knowledge acquisition and confidence and possible improvements in mental well-being.

To our knowledge, this is the first study to assess the unmediated web-based conversations of mothers during the first year of the postpartum period. Our findings portray parental experiences and perceptions without the influence of researchers or controlled research settings [36]. This design enables insight into the communication and output of emotions that women may experience at any time of the day, which may be lost to recall or have subsided when sought in a research or clinical setting.

### Limitations

The following limitations should be considered while interpreting these findings. Anonymized data were interrogated, and therefore, demographic and geographical user information could not be obtained. User demographics may influence engagement with the forum as well as postpartum needs and experiences. Although users cannot be demographically profiled, the anonymous nature of the forum allows for uninhibited discussion, providing rich data on the needs of participating women. Due to site management and restrictions relating to the seeking or provision of medical advice via the forum, posts may have been deleted before data were collected.

### Conclusions

The postpartum period involves a major life transition that requires increased levels of social, emotional, and health professional support. Our results demonstrate that engagement in web-based forums to seek help and support during the postpartum period is common, with infant health and well-being being primary concerns for new mothers during this time. A lack of discussion about maternal health was observed, emphasizing the need for improved awareness and novel engagement strategies. The identification of a maternal learning cycle at play within the forum demonstrates the significant role of web-based communities in maternal social support and in defining parenting. Further exploration is needed to understand how health care professionals can provide targeted and personalized support to women in postpartum period, where infant needs are prioritized above their own, particularly for those experiencing increased levels of distress and uncertainty.

## Appendix

Multimedia Appendix 1 Frequently used words in forum user-generated content (word cloud).
