# Supplementary figures and images for: Support Seeking in the Postpartum Period: Content Analysis of Posts in Web-Based Parenting Discussion Groups
Source: J Med Internet Res. 2021 Jul 15;23(7):e26600. doi: 10.2196/26600 (PMC8323017; doi:10.2196/26600)

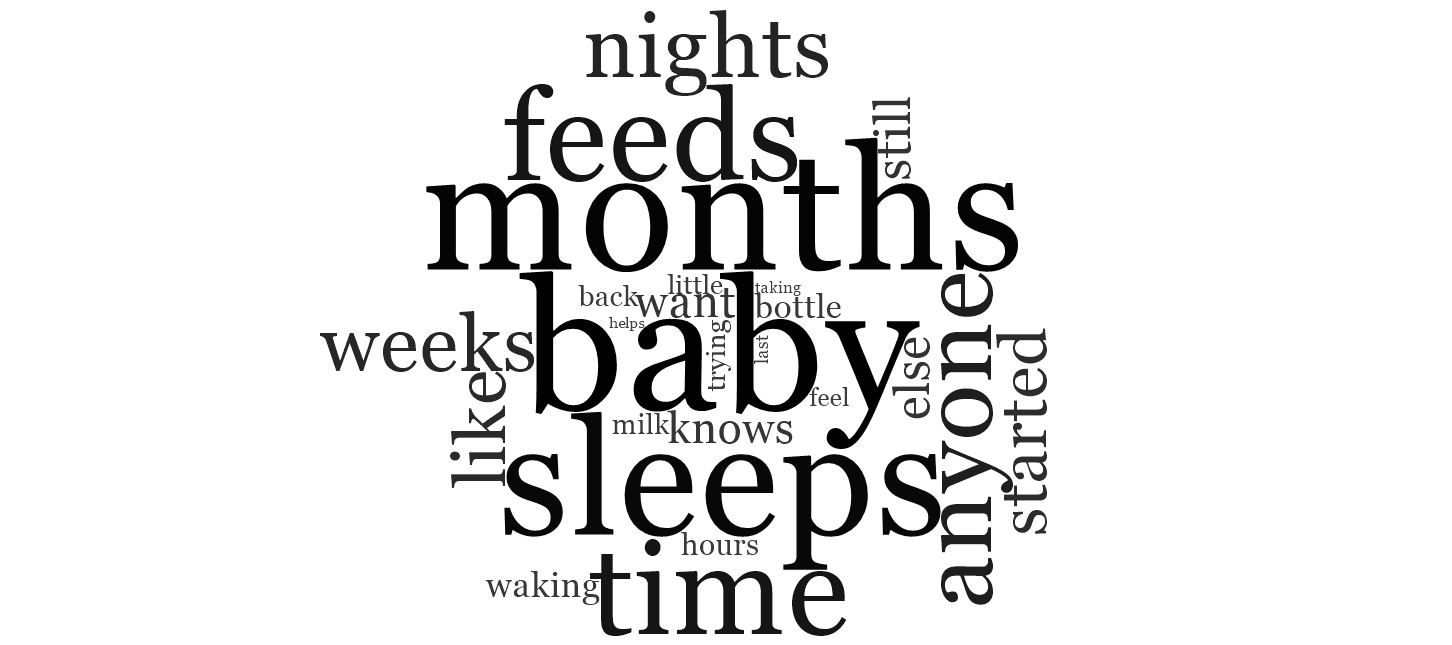

Supplement: Multimedia Appendix 1 [file jmir_v23i7e26600_app1.png]
